# NESARC Findings on Alcohol Abuse and Dependence

**Published:** 2006

**Authors:** Raul Caetano

**Affiliations:** Raul Caetano, M.D., M.P.H., Ph.D., is a professor and dean of the Dallas Regional Campus, University of Texas Health Science Center School of Public Health, Dallas, Texas

**Keywords:** National Epidemiologic Survey on Alcohol and Related Conditions (NESARC), survey, general population survey, statistical data, epidemiology, United States, alcohol abuse, alcohol dependence, alcohol use disorders, drinking patterns, comorbidity, treatment, recovery

## Abstract

Epidemiology is one of the central disciplines of public health. Its aim is to determine how prevalent a disease is within a population and to identify people who may be at particular risk for it. Epidemiological data provide information that help researchers, public health professionals, and treatment providers alike to better understand the course of disease and to improve its treatment. The National Epidemiologic Survey on Alcohol and Related Conditions (NESARC) is an example of a large, random, representative survey of people living in the United States. This survey addressed all aspects of alcohol use—from determining when a respondent took his or her first drink to discovering whether he or she has experienced co-occurring mental health problems. NESARC’s data have several practical applications: to help us to better define the intricate relationship between alcohol use and comorbidity, understand high-risk drinking patterns, design better-targeted treatment approaches, and monitor recovery from alcohol use disorders. Analyses with NESARC data have only just begun. As more researchers take advantage of the richness of this data set, more knowledge will be gained, helping to advance treatment interventions in the alcohol field.

Epidemiology is one of the central disciplines of public health. Its aim is to provide information on the distribution of disease and the risk factors for it within populations. For treatment providers, epidemiological data provide increased understanding of the natural history of disease and help improve treatment; for public health professionals, these studies help identify populations or population subgroups at greatest risk for a particular disease or diseases. Using the results of epidemiological studies, health care professionals can tailor prevention efforts to the risk factors for the diseases of interest and direct these efforts toward the population groups at highest risk of suffering from them.

The National Epidemiologic Survey on Alcohol and Related Conditions (NESARC), upon which the articles in this issue of *Alcohol Research & Health* are based, is a general population survey, that is, a study in which the subjects are a random, representative sample of a larger population. The main advantage of general population surveys is that as long as the subjects in the study are selected at random, the findings from the study are generalizable to the larger population, independent of the study sample size. Sample size matters, however, because the larger the sample size, the more accurate (i.e., precisely representative) the findings will be. NESARC successfully fulfills these two conditions: random selection of subjects and large sample size. Respondents were selected at random from the U.S. household population age 18 or older, and the sample size is large—43,093 respondents. Thus, results from NESARC are accurate and can be generalized to the larger U.S. population.

NESARC, like many large surveys, was designed to answer a variety of research questions. For instance, Grant and colleagues (2004*a*) determined the prevalence of alcohol abuse and dependence in the U.S. population in the years the NESARC data were collected, 2001–2002. When these results are compared with findings from a previous large survey conducted in 1991–1992 (the National Longitudinal Alcohol Epidemiologic Survey, or NLAES), we not only can determine the latest prevalence of alcohol use disorders in the United States, but also track any changes that may have occurred over the past 10 years.

These findings can be used in important ways to respond to alcohol use disorders (AUDs) in the United States. The NESARC data underscore the need to direct prevention approaches toward the young (ages 18 to 29) and to develop culturally appropriate prevention messages tailored to groups with high or growing prevalence of AUDs. The NESARC prevalence data also provide treatment program administrators with information on the characteristics of the populations they should be prepared to receive and for whom they should tailor interventions.

Other NESARC findings have described the association between alcohol and drug use disorders (Stinson et al. 2005), alcohol and drug use disorders and personality disorders (Grant et al. 2004*b*), and alcohol and drug use disorders and mood and anxiety disorders (Grant et al. 2004*c*). In general, these studies show that alcohol abuse and dependence frequently co-occur with other psychiatric disorders. People who seek treatment for an AUD are even more likely than others to present with a mood or anxiety disorder.

## Putting NESARC Into Practice

What do the findings from the NESARC data set mean for people working in the prevention and treatment fields?

### Comorbidity

NESARC offers us an opportunity to examine in detail comorbidity between AUDs and other psychiatric problems. For instance, NESARC data enable us to distinguish independent mood and anxiety disorders from those that are alcohol and other drug–induced, and they document the use of alcohol and drugs for relief of symptoms of mood and anxiety disorders. This can help to identify the proportion of the population for which treatment of AUDs would not necessarily relieve mood and anxiety problems, as well as the proportion that may be drinking to self-medicate mood and anxiety disorders. Understanding the complexities of comorbidity has particular relevance for people designing successful prevention approaches.

**Frequently Asked Questions From NESARC Data Users**

Since the data from NESARC were first made available to the public on the NESARC Web site (http://niaaa.census.gov/), public data users have made many inquiries about the data. Below are a few of the most frequently asked questions. Users who require more information or who need help accessing the data should contact the NESARC contact person, Ms. Nekisha Lakins, at nlakins@csrincorporated.com.

***Q: Is the data set available in alternative data formats?***

A: The data set currently is available in flat text format and can be downloaded from the NESARC Web site along with a coding file that will read the text file into a SAS data file. Other commonly used statistical software packages, such as STATA and SPSS, can read SAS data files and convert them into their respective data formats. STATA and SPSS users may contact the NESARC contact person to obtain the NESARC data set in SAS format and information on how to access it.

***Q: Are additional geographic identifiers (e.g., county) available on request?***

A: NESARC is designed to generate nationally representative estimates, although the data set includes State FIPS code. To ensure the confidentiality of all respondents, lower level geographic information is not available to any users.

***Q: Are additional data available on request?***

A: The public data file posted on the Web site is the complete data set. There are no supplementary data sets containing additional variables.

***Q: Can other data sets be linked to the NESARC data set?***

A: The NESARC data set stands alone and does not contain the information necessary to link it to other data sets.

***Q: When will data from Wave 2 of NESARC be available to public users?***

A: The Wave 2 interviews were completed in 2005. Data from Wave 2 are expected to be released in summer 2007.

***Q: Are the specific algorithms used to create the psychological diagnoses available to the public?***

A: All diagnoses in Wave 1 of NESARC were made according to the *Diagnostic and Statistical Manual of Mental Disorders, Fourth Edition* (DSM–IV). This reference should help data users understand how the diagnoses were constructed.

***Q: Where can I obtain findings and statistics from NESARC?***

A: A small subset of articles based on the NESARC data are published in this issue of *Alcohol Research & Health*. A full list of NESARC publications can be found on the NESARC Web site. The Web site also includes the new NIAAA Alcohol Epidemiologic Data Reference Manual, *Alcohol Use and Alcohol Use Disorders in the United States: Main Findings from the 2001–2002 National Epidemiologic Survey on Alcohol and Related Conditions (NESARC)*. This manual presents information in table form on the prevalence and patterns of alcohol use, DSM–IV alcohol abuse and dependence, family history of alcoholism, problems associated with alcohol, alcoholism treatment, drug and nicotine use disorders, mood and anxiety disorders, personality and conduct disorders, pathological gambling, and a number of alcohol-related physical conditions. Hard copies of the manual can be ordered on NIAAA’s Web site or through the NESARC contact person.

The strong association between AUDs and other psychiatric diseases indicates that prevention strategies addressing only one problem may not be as effective as those targeting comorbid conditions. These findings may be especially relevant for designing individually based prevention strategies, because these treatments so often are based on providing accurate educational information about the dangers associated with drinking and the potential reasons for alcohol abuse and dependence.

The results in NESARC also show that the occurrence of comorbid psychiatric disorders with AUDs is not rare but happens in a quarter to almost half of the clients in treatment. It is therefore imperative that alcoholism treatment providers be trained to identify the signs and symptoms of these comorbid conditions as well as to have information about referral patterns for these people. Thus, if a provider who is not a medical doctor recognizes a comorbid mood disorder in a client, that provider will be able to refer the client to a mental health professional who then can confirm the diagnosis and prescribe medication if appropriate.

Treatment plans for clients presenting with comorbid conditions also must be individualized, taking into consideration the nature and severity of the co-occurring disorder. These plans may call for longer periods of treatment, residential rather than outpatient treatment, prescription of anxiolytic or antidepressive medication in addition to group therapy or one-on-one discussions, or entirely different approaches in addressing clients’ excessive alcohol use. Evidence already exists that addressing the social and psychiatric needs of alcohol-dependent patients improves alcohol outcomes (McLellan et al. 1993, 1998; Ray et al. 2005). Taking into consideration co-occurring disorders will thus help providers adapt their expectations about clients’ recovery processes. A client’s course of dependence, and perhaps response to treatment, may be changed by the co-occurring disorder. For instance, clients with AUDs that co-occur with mood disorders may recover much faster than other clients if they receive adequate anti-depressant treatment.

**NESARC’s Key Goals**

- To determine the extent of alcohol use disorders (AUDs) and their associated disabilities in the general population
- To estimate the size, characteristics, and time trends of populations of special concern, including alcohol abusers and people in the general population otherwise impaired by the use of alcohol
- To provide more complete recording and tabulation of AUDs and their associated disabilities in important subgroups of the population
- To estimate changes over time in AUDs and their associated disabilities and to identify factors that impact their remission, chronicity, stability, and initiation
- To increase our understanding of the natural history of AUDs and their associated disabilities
- To determine the number of individuals seeking and receiving treatment through alcoholism treatment programs and services, including those not otherwise represented in periodic surveys of treatment facilities or populations in treatment
- To determine the demographic characteristics of people seeking and receiving treatment through alcoholism treatment programs and services, including those not otherwise represented in surveys of treatment facilities or populations in treatment
- To measure the number of people in the population in need of but not currently receiving treatment for alcoholism, and their associated disabilities
- To provide information concerning barriers to alcohol-related treatment services, particularly among low-income groups, women, young adults, and minorities
- To determine the economic impact of AUDs and their associated disabilities on productivity in the workplace
- To determine the magnitude and extent of binge drinking among college-aged young adults and to identify their characteristics and the risk factors that influence the initiation and remission of this hazardous drinking pattern
- To determine the boundaries between safe and hazardous drinking levels and patterns for various types of AUDs and their associated medical, social, and psychological sequelae
- To determine the associations between AUDs and their major physical and mental disabilities
- To measure disability as a separate and distinct dimension of treatment need
- To determine the extent of major alcohol-related mental and physical disabilities that are substance-induced disorders and differentiate those substance-induced disorders from disorders reflecting true independent mental conditions.

### High-Risk Drinking Patterns

NESARC questions were explicitly designed to measure overall alcohol consumption as well as the prevalence of heavy episodic (or binge) drinking. This information has particular relevance for professionals designing prevention measures because it allows for the identification of population subgroups that should be a target for prevention approaches to avoid acute and chronic problems associated with heavy episodic drinking. Alcohol-related problems such as injuries and drinking and driving occur not only among chronic drinkers who are alcohol dependent but also among drinkers who may not be alcohol dependent but who engage in heavy episodic drinking.

### Treatment

As mentioned above, NESARC results on comorbidity between AUDs and psychiatric disorders are important for treatment providers when designing provider training programs and planning treatment. Other NESARC results about the prevalence of AUDs in the population can help treatment providers target their programs to the populations they will serve. For example, NESARC information on trends of abuse and dependence over time helps identify populations that may be at particular risk for alcohol-related problems. By alerting providers to the needs of these special populations, data on the prevalence of alcohol dependence among Blacks and Hispanics and other ethnic minority groups could lead to better-targeted treatment for these special populations.

### Recovery

Recovery from alcohol dependence is another topic that has been investigated in NESARC. Dawson and colleagues (2005) examined the percentage of individuals with “prior to past year” (PPY) alcohol dependence who recovered in a 12-month span as well as the factors associated with their recovery. The findings suggest that a substantial number of people recover from alcohol dependence in spite of its chronic characteristics. This should provide a positive incentive for people seeking treatment and for providers working with these clients. Second, a considerable number of people appear to recover without professional intervention. Further understanding of this finding is needed to identify people who tried to get into treatment and failed to gain access, people who received a type of treatment that was not recognized in the survey, and those who genuinely recovered on their own without trying to obtain treatment. Knowing more about each of these groups and their trajectories out of dependence may help in designing recovery programs. Third, some people seem able to go back to drinking without experiencing their previous alcohol-related problems. This result too needs further research so that these individuals can be clearly identified and this potential course of recovery can be considered.

## Wave 2

The examples above are just a few of the many scientific findings that have come out of analyses of the NESARC data. More data are being added to this survey. In 2004 to 2005, Wave 2 data were collected, during which every effort was made to reinterview all 43,093 of the respondents from Wave 1. Wave 2 data expand the NESARC data set even further, adding information on incidence, recurrence, and remission of alcohol and drug use disorders and mental health disorders. As shown in the accompanying textbox, NESARC findings set the stage for a variety of investigations, and as more researchers take advantage of the richness of this data set, more knowledge will be gained to help advance prevention and treatment interventions in the alcohol field.
